# Supplementary material for: Roles of Tubulin Concentration during Prometaphase and Ran-GTP during Anaphase of Caenorhabditis elegans Meiosis
Source: Life Sci Alliance. 2024 Jul 3;7(9):e202402884. doi: 10.26508/lsa.202402884 (PMC11222656; doi:10.26508/lsa.202402884)
Supplement: Supplementary file 11 [file LSA-2024-02884_TableS1.docx]

Table S1: C. elegans strains used in this study.

| Strain name | Genotype | Source |
| --- | --- | --- |
| FM917 | *fxIs1 [pie-1p::TIR1::mRuby, I:2851009] I;*  *ltIs37 [pAA64; pie-1p::mCh::his-58 + unc-119(+)];*  *ruIs57 [pie-1p::GFP::tubulin + unc-119(+)] V* | This study |
| FM1054 | *fxIs1[pie-1p::TIR1::mRuby, I:2851009] I;*  *ran-3(syb7781[ ran-3-3xGAS-AID-3xGAS-HALO]) II; ltIs37 [pAA64; pie-1p::mCh::his-58 + unc-119(+)];*  *ruIs57 [pie-1p::GFP::tubulin + unc-119(+)] V* | This study |
| FM1056 | *fxIs1[pie-1p::TIR1::mRuby, I:2851009] I;*  *ran-2(syb7819[ran-2-3xGAS-AID-3xGAS-HALO]) III; ltIs37 [pAA64; pie-1p::mCh::his-58 + unc-119(+)];*  *ruIs57 [pie-1p::GFP::tubulin + unc-119(+)] V* | This study |
| FM717 | *bus-17(e2800)X;*  *ltSi1412 [pNA20; Pmex-5::mNeonGreen::tbb-2 operon linker mCh::his-11::Ptbb-2; cb-unc-199(+)]I;*  *unc-119(ed3)III clone B (MOS I insertion)* | This study |
| NM5402 | *jsSi1579 [loxP::rpl-28p::FRT::GFP::his-58 FRT3] II. bqSi711 [mex-5p::FLP::SL2::mNG + unc-119(+)] IV.* | Gifted from Nonet Lab |
| FM971 | *duSi18[GFP(SMU)-GCN4-pLI] II;*  *ltIs37 [pAA64; pie-1p::mCh::his-58 + unc-119(+)];*  *him-8(e1489)* | This study |
| FM1011 | *duSi20[GFP(SMU)::tba-2(T349E)] II*  *ltIs37 [pAA64; pie-1p::mCh::his-58 + unc-119(+)];*  *him-8(e1489)* | This study |
| FM628 | *unc-119(ed3) III;*  *ltSi464[pNH103; Pmex-5::npp6::GFP::tbb-2 3'UTR; cbunc-119(+)] I;*  *ItIs37[pAA64; pie-1::mCherry::his-58; unc-119 (+)] IV* | A gift from Oegema-Desai Lab |
| BN359 | *ima-2(ok256) I/hT2[bli-4(e937) let-?(q782) qIs48] (I;III); qaIs3502[pie-1p::YFP::lmn-1 + pie-1p::CFP::H2B + unc-119(+)]* | CGC |
| FM991 | *wjIs76[Cn_unc-119(+); pie-1p::mKate2::tba-2];*  *vit-2(crg9070[vit-2::gfp]) X;*  *egxSi126 [mex-5p::hsp-3(aa1-19)::halotag::HDEL::pie-1 3’UTR+ unc-119(+)] I. "* | This study |
| FM691 | *cox-4(zu476[cox-4::eGFP::3xFLAG]) I;*  *wjIs76[Cn_unc-119(+); pie-1p::mKate2::tba-2]* | This study |
| CZ18550 | *juSi123[rpl-29::GFP] II; rpl-29(tm3555) IV* | CGC |
| ABR5 | *staIs1 [pie-1p::GFP + unc-119(+)];*  *unc-119(ed3) III* | CGC |
| FM1103 | *duSi21[HALO(smu)] II;*  *ruIs57 [pie-1p::GFP::tubulin + unc-119(+)] V*  *itIs37 [pie-1p::mCh::H2B::pie-1 3'UTR + unc-119(+)] IV"* | This study |
| FM1168 | *duSi23[minus7-GFP(SMU)::GCn4-pLI] II;*  *ItIs37[pAA64; pie-1::mCherry::his-58; unc-119 (+)] IV* | Negative Charge; this study |
| FM1169 | *duSi24[plus7-GFP(SMU)::GCn4-pLI] II;*  *ItIs37[pAA64; pie-1::mCherry::his-58; unc-119 (+)] IV* | Neutral Charge; this study |
| FM1180 | *duSi25[plus21-GFP(SMU)::GCn4-pLI] II;*  *ItIs37[pAA64; pie-1::mCherry::his-58; unc-119 (+)] IV* | Positive charge; this study |
