# Supplementary material for: Roles of Tubulin Concentration during Prometaphase and Ran-GTP during Anaphase of Caenorhabditis elegans Meiosis
Source: Life Sci Alliance. 2024 Jul 3;7(9):e202402884. doi: 10.26508/lsa.202402884 (PMC11222656; doi:10.26508/lsa.202402884)
Supplement: Supplementary file 12 [file LSA-2024-02884_Supplemental_Data_1.docx]

ran-3(syb7781) II:

GGAAAAGTGTTTGCTATGGGAAAGAACACAGACAATGCTCTCGGCCTCGGTAATTGGACTGGAAAGGACGACCAACAGCATTGGTTGTACGATACAATCCAGGAAATAGAATTCGATTCGAAGATCGTTGGTGTTTCTGCCAAACTAGCCACTTCTATCGCCTGGTCTGAGGATGGAACCGCCTACGCTTGGGGTTTTGATACTACCGGACAACTTGGTCTCGGATTGAAAGACGAAGACGAGAAGgtaatttttcaaaactcaaactatcaataaataaaatattcaatcttattttccagATGGTGTCCAAGCCAGAGGAGATCAGCTCC**GCACAC**CTTGACGGTTAT**AGT**ATTATCGGGGCTTCGATTTCCGATCAGCACACTTTGATTATTGCCAAGAAAAATGGAGCATCGGGAGCCTCAGGAGCATCGATGCCTAAAGATCCAGCCAAACCTCCGGCCAAGGCACAAGTTGTGGGATGGCCACCGGTGAGATCATACCGGAAGAACGTGATGGTTTCCTGCCAAAAATCAAGCGGTGGCCCGGAGGCGGCGGCGTTCGTGAAGGGAGCATCGGGAGCCTCAGGAGCATCGATGGCTGAAATTGGCACAGGATTCCCGTTTGACCCCCACTACGTCGAGGTCCTCGGAGAGCGTATGCACTACGTCGACGTCGGACCACGTGACGGAACCCCAGTCCTCTTCCTCCACGGAAACCCAACCTCCTCCTACGTCTGGCGTAACATCATCCCACACGTCGCCCCAACCCACCGTTGCATCGCCCCAGACCTCATCGGAATGGGAAAGTCCGACAAGCCAGACCTCGGATACTTCTTCGACGACCACGTCCGTTTCATGGACGCCTTCATCGAGGCCCTCGGACTCGAGGAGGTCGTCCTCGTCATCCACGACTGGGGATCCGCCCTCGGATTCCACTGGGCCAAGCGTAACCCAGAGCGTGTCAAGgtaagtttaaacatatatatactaactaaccctgattatttaaattttcagGGAATCGCCTTCATGGAGTTCATCCGTCCAATCCCAACCTGGGACGAGTGGCCAGAGTTCGCCCGTGAGACCTTCCAAGCCTTCCGTACCACCGACGTCGGACGTAAGCTCATCATCGACCAAAACGTCTTCATCGAGGGAACCCTCCCAATGGGAGTCGTCCGTCCACTCACCGAGGTCGAGATGGACCACTACCGTGAGCCATTCCTCAACCCAGTCGACCGTGAGCCACTCTGGCGTTTCCCAAACGAGCTCCCAATCGCCGGAGAGCCAGCCAACATCGTCGCCCTCGTCGAGGAGTACATGGACTGGCTCCACCAATCCCCAGTCCCAAAGCTCCTCTTCTGGGGAACCCCAGGAGTCCTCATCCCACCAGCCGAGGCCGCCCGTCTCGCCAAGTCCCTCCCAAACTGCAAGgtaagtttaaacagttcggtactaactaaccatacatatttaaattttcagGCCGTCGACATCGGACCAGGACTCAACCTCCTCCAAGAGGACAACCCAGACCTCATCGGATCCGAGATCGCCCGTTGGCTCTCCACCCTCGAGATCTCCGGATAAattatttggttttattctcaacttttatatcagttttgttttgtctctgtagcattattttgttattttttcgtttcccggtagccaattcgattgttctccagtaacattctcatcaatttttctgttttttttatcatttcatttttgtcaagtagcatcagtcatgtaagaaagggatagagttcccttctgtgaaaatggagaattgttgaaacgtcgttgcacaacgacgtttaaacacttcactctatccctggattgatttccaacttctatgttttctcaaaaccccctattagttctgctatatattggaatccaaaaatttttcatttttagcctgagttatgttgttctcttatcatgtgaactcactgttttataatccgtttcaccatttatggtaaacgtttcctgg

3’ of ran-3 are highlighted in yellow (within which synonymous mutation is labeled in blue text); AID in red text followed by halo sequence in blue text. Linker sequences (in purple) are inserted among the genes.

ran-2(syb7819) III:

AGCTATTGAAGTTGCAGgtaagaaatataaaaatattttaatataactactttcaatttttaagAAAATATCGTCCGCCGAGTGGAGTCTGTCAAGCGTAACCCGATTCCGGCCACAACTCAATTAGTTAACAATATTGTTGCTCAATGTGCAGGAACAGGAGTTAAGgtaggtatttttcaagcttattctaaaaaacgtttaatatatgagacagactttacagGCTGAAACTGATTGGGGATATGGTGCCGATCCACAAGTGATTTCACGTTTGTTCTCGGAACTTGTTGCTCGCGGCCATTTCAAG**CTT**GAGCTGGCTCTCCTTCAACGCTTTTTCgtaagtctcacaactatattttatggttatttttttcaatttttcagCCTTCACAAGGAGCATCGGGAGCCTCAGGAGCATCGATGCCTAAAGATCCAGCCAAACCTCCGGCCAAGGCACAAGTTGTGGGATGGCCACCGGTGAGATCATACCGGAAGAACGTGATGGTTTCCTGCCAAAAATCAAGCGGTGGCCCGGAGGCGGCGGCGTTCGTGAAGGGAGCATCGGGAGCCTCAGGAGCATCGATGGCTGAAATTGGCACAGGATTCCCGTTTGACCCCCACTACGTCGAGGTCCTCGGAGAGCGTATGCACTACGTCGACGTCGGACCACGTGACGGAACCCCAGTCCTCTTCCTCCACGGAAACCCAACCTCCTCCTACGTCTGGCGTAACATCATCCCACACGTCGCCCCAACCCACCGTTGCATCGCCCCAGACCTCATCGGAATGGGAAAGTCCGACAAGCCAGACCTCGGATACTTCTTCGACGACCACGTCCGTTTCATGGACGCCTTCATCGAGGCCCTCGGACTCGAGGAGGTCGTCCTCGTCATCCACGACTGGGGATCCGCCCTCGGATTCCACTGGGCCAAGCGTAACCCAGAGCGTGTCAAGgtaagtttaaacatatatatactaactaaccctgattatttaaattttcagGGAATCGCCTTCATGGAGTTCATCCGTCCAATCCCAACCTGGGACGAGTGGCCAGAGTTCGCCCGTGAGACCTTCCAAGCCTTCCGTACCACCGACGTCGGACGTAAGCTCATCATCGACCAAAACGTCTTCATCGAGGGAACCCTCCCAATGGGAGTCGTCCGTCCACTCACCGAGGTCGAGATGGACCACTACCGTGAGCCATTCCTCAACCCAGTCGACCGTGAGCCACTCTGGCGTTTCCCAAACGAGCTCCCAATCGCCGGAGAGCCAGCCAACATCGTCGCCCTCGTCGAGGAGTACATGGACTGGCTCCACCAATCCCCAGTCCCAAAGCTCCTCTTCTGGGGAACCCCAGGAGTCCTCATCCCACCAGCCGAGGCCGCCCGTCTCGCCAAGTCCCTCCCAAACTGCAAGgtaagtttaaacagttcggtactaactaaccatacatatttaaattttcagGCCGTCGACATCGGACCAGGACTCAACCTCCTCCAAGAGGACAACCCAGACCTCATCGGATCCGAGATCGCCCGTTGGCTCTCCACCCTCGAGATCTCCGGATAAtcgagcttctctacacgatatcccagtcttcgcgatttttttacatgatttcagataagccgtggttattttatatttggatctacaatacatgtattattcatcgtatcgtgaacaatatatttcttcaacttccaatgtatacacgagttatcaatttgttttaattttgtttctctaatttccactaatttttagtttaatactttaaatctctttcttgtacgtgtaaatgcttcaatccgtttttcaagtaaatttttgtgaacgaagttgtttttatgattacatgttttatgtctttgtgaactttttt

3’ of ran-2 are highlighted in yellow (within which synonymous mutation is labeled in blue text); AID in red text followed by halo sequence in blue text. Linker sequences (in purple) are inserted among the genes.
