# Supplementary material for: Roles of Tubulin Concentration during Prometaphase and Ran-GTP during Anaphase of Caenorhabditis elegans Meiosis
Source: Life Sci Alliance. 2024 Jul 3;7(9):e202402884. doi: 10.26508/lsa.202402884 (PMC11222656; doi:10.26508/lsa.202402884)
Supplement: Supplementary file 13 [file LSA-2024-02884_Supplemental_Data_2.docx]

1. Sequence of GFP::GCN4-pLi:

ATGCCT**GCATcc**TCCAAGGGAGAGGAGCTCTTCACCGGAGTCGTCCCAATCCTCGTCGAGCTCGACGGAGgtattttcctgcatttttcaactgggaaaatgaaagaaaatcgataatttcagACGTCAACGGACACAAGTTCTCCGTCTCCGGAGAGGGAGAGGGAGACGCCACCTACGGAAAGCTCACCCTCAAGTTCATCTGCACCACCGGAAAGCTCCCAGTCCCATGGCCAACCCTCGTCACCACCTTCTGCTACGGAGgtaagatatgggaaagaaggaaaaaaccgagattttacttgaaaaattgaatttttcgcgggattttcaccaaaaattgttgaatattcattatttcacgctgtaaaacaaaaaaaaaaaaaatcaaaaactacgttgaaatcgcgtttttaagcgaattttcttcagaattgccagattttaaccccaaattttgcagtttttaaataaaatttcaccttttcggctcaaattgtagattttcctgaaaatttagtacaaaaaacaatttcctcgtaaatttttcaaattttcagTCCAATGCTTCTCCCGTTACCCAGACCACATGAAGCGTCACGACTTCTTCAAGTCCGCCATGCCAGAGGGATACGTCCAAGAGCGTACCATCTTCTTCAAGGACGACGGAAACTACAAGACCCGTGCCGAGGTCAAGTTCGAGGGAGACACCCTCGTCAACCGTATCGAGCTCAAGGGAATCGACTTCAAGGAGGACGGAAACATCCTCGGACACAAGCTCGAGTACAACTACAACTCCCACAACGTCTACATCATGGCCGACAAGCAAAAGAACGGAATCAAGGTCAACTTCAAGgtacggattgaaattgctttaaaatttgaaaaattgattaaaaagtgcattttttaagctttgaccgacttaaaattagatttctgagcctattttctgagaattggaatttttttcattgtgaaagttcaaagaatagcgtgaatgattagaaaatattgtaaaatttcaattttttcctataaaaaaggatttttttaggaatcaaaaattgcaaaatgatgccctaaaattcgaaaataaatataaaaattggcggtttctcaaaaatctagaattccgaccttaattattaatttttttacaatatttttttttgaaaaaaatccagaaaatttgaatttcgtagtttgtagtctaggcctctatcaattaaattttcgattttttgagtaaaatttcgaatttactattatttggaccaaaattgtatttttttttcagaattaaatttataaaattttcagaaattaaatttataaaaaaaaaaaaaaattaaaaaaaaaaaaaaaaaattaaaaattaaaaattaaaaaattaagttttttttactcaaaattttgcactgaaattcgaaaatctaaaaatccgacctaaagtctgtattttttcaacaaaaattcagaaaaaactcaaaaacttgtattttgtagccagtcaaccactttctaaaatatcaaatttgaatttttcaagcatttttacattgaaaaatctaatttttcgagtgaaaccacttgaaaatctgaaaattgaattaatttctgaactttagagattttttgtcaaaattttagataaagggatttttaaacaaaaaattgattttttaatcgaaaaatactgggatttatgggttttttaaaagaaaaacgggggtttgaatgaaaaatcgcctgaaatcttagaaaattaattaaaaaactatgatttaattccaaaaattaccgaaaatatcaaattttccatttttaaaccctaaattctttcagATCCGTCACAACATCGAGGACGGATCCGTCCAACTCGCCGACCACTACCAACAAAACACCCCAATCGGAGACGGACCAGTCCTCCTCCCAGACAACCACTACCTCTCCACCCAATCCGCCCTCTCCAAGgtagattttttagaatttttgggtttttgaagtagaaaatcataaaaatctagggtttttatgaattgttttgaagaaaaattgcaaaaattccacaaaatggaagaaaaataactttggaagcgcatttttcgcaaaaaaaccgaaatttttgcgtaaaatttcaaaattgcaataaaaattccacaaacatcaaatttcttaaattttttataaaaaattggatggaaacactcttgaatttagaaaaaaaatcagtttttctcatctaaaacttcaaaatttcggtgtaatcccattaaaattgccacaaaattcggaaatttcacctgaaatagagtgaataatttaaaatgttcagaaattcatattttgtcattttaaaagcattaaaacaaatcaaaaatctattttttttggttggaaaagttcaaaattctggagaaattacattaaaattttcgtaaaatatggtaaataaacgaaaatgttgagaaattaaagaaaagttacaatttttagcttaaaaattcaacattttgaggaaatgccacctaaaaaagtgactaatcgaaaatgttgagaaattaaaattgccaccatttatttatataaactactctaaaattacaattttcatgttaaaaattaataaaaaatctactttttccaaactacagtaaccttaccgtatacctacagtacctgaacattgccccccaccagctcccaacccaatacctcctcaaaaacttacacctcaatttttcataaactacagtaaccctaccaaaaaagcacaaaaaaaaatctacattcattttccaacaattttcaatattttcagGACCCAAACGAGAAGCGTGACCACATGGTCCTCCTCGAGTTCGTCACCGCCGCCGGAATCACCCACGGAATGGACGAGCTCTACAAGCCTGCATCaGGAGGcTCCGGtGGgTCgGGtGGcTCaGGgGGAATGAAACAGATAGAGGATAAGCTTGAGGAGATTCTGTCCAAGCTGTATCACATTGAGAATGAGCTCGCTCGCATCAAGAAGCTCTTGGGGGAGCGGTAA

GFP optimized for germline expression is highlighted in green. GCN4-pLi is colored red.

2. Sequence of GFP::GCN4-pLi with negative charge:

**“GAAGATGAAGATGAGGACGAGGCA”** was inserted onto GFP::GCN4-pLi before GCATcc (highlighted in bold)

3. Sequence of GFP::GCN4-pLi with neutral charge:

**“CGCAGACGTAGACGTCGTCGAGCAGCT”** was inserted onto GFP::GCN4-pLi before GCATcc (highlighted in bold)

4. Sequence of GFP::GCN4-pLi with positive charge:

**“CGCAGACGTAGACGTCGTCGAGCACGTCGTCGAGCACGTCGACGTGCCCGTGCTCGTCGTCGTCGAGCACGTCGTCGAGCA”** was inserted onto GFP::GCN4-pLi before GCATcc (highlighted in bold)

5. Sequence of GFP::tba-2(T349E):
ATGCCTGCATccTCCAAGGGAGAGGAGCTCTTCACCGGAGTCGTCCCAATCCTCGTCGAGCTCGACGGAGgtattttcctgcatttttcaactgggaaaatgaaagaaaatcgataatttcagACGTCAACGGACACAAGTTCTCCGTCTCCGGAGAGGGAGAGGGAGACGCCACCTACGGAAAGCTCACCCTCAAGTTCATCTGCACCACCGGAAAGCTCCCAGTCCCATGGCCAACCCTCGTCACCACCTTCTGCTACGGAGgtaagatatgggaaagaaggaaaaaaccgagattttacttgaaaaattgaatttttcgcgggattttcaccaaaaattgttgaatattcattatttcacgctgtaaaacaaaaaaaaaaaaaatcaaaaactacgttgaaatcgcgtttttaagcgaattttcttcagaattgccagattttaaccccaaattttgcagtttttaaataaaatttcaccttttcggctcaaattgtagattttcctgaaaatttagtacaaaaaacaatttcctcgtaaatttttcaaattttcagTCCAATGCTTCTCCCGTTACCCAGACCACATGAAGCGTCACGACTTCTTCAAGTCCGCCATGCCAGAGGGATACGTCCAAGAGCGTACCATCTTCTTCAAGGACGACGGAAACTACAAGACCCGTGCCGAGGTCAAGTTCGAGGGAGACACCCTCGTCAACCGTATCGAGCTCAAGGGAATCGACTTCAAGGAGGACGGAAACATCCTCGGACACAAGCTCGAGTACAACTACAACTCCCACAACGTCTACATCATGGCCGACAAGCAAAAGAACGGAATCAAGGTCAACTTCAAGgtacggattgaaattgctttaaaatttgaaaaattgattaaaaagtgcattttttaagctttgaccgacttaaaattagatttctgagcctattttctgagaattggaatttttttcattgtgaaagttcaaagaatagcgtgaatgattagaaaatattgtaaaatttcaattttttcctataaaaaaggatttttttaggaatcaaaaattgcaaaatgatgccctaaaattcgaaaataaatataaaaattggcggtttctcaaaaatctagaattccgaccttaattattaatttttttacaatatttttttttgaaaaaaatccagaaaatttgaatttcgtagtttgtagtctaggcctctatcaattaaattttcgattttttgagtaaaatttcgaatttactattatttggaccaaaattgtatttttttttcagaattaaatttataaaattttcagaaattaaatttataaaaaaaaaaaaaaattaaaaaaaaaaaaaaaaaattaaaaattaaaaattaaaaaattaagttttttttactcaaaattttgcactgaaattcgaaaatctaaaaatccgacctaaagtctgtattttttcaacaaaaattcagaaaaaactcaaaaacttgtattttgtagccagtcaaccactttctaaaatatcaaatttgaatttttcaagcatttttacattgaaaaatctaatttttcgagtgaaaccacttgaaaatctgaaaattgaattaatttctgaactttagagattttttgtcaaaattttagataaagggatttttaaacaaaaaattgattttttaatcgaaaaatactgggatttatgggttttttaaaagaaaaacgggggtttgaatgaaaaatcgcctgaaatcttagaaaattaattaaaaaactatgatttaattccaaaaattaccgaaaatatcaaattttccatttttaaaccctaaattctttcagATCCGTCACAACATCGAGGACGGATCCGTCCAACTCGCCGACCACTACCAACAAAACACCCCAATCGGAGACGGACCAGTCCTCCTCCCAGACAACCACTACCTCTCCACCCAATCCGCCCTCTCCAAGgtagattttttagaatttttgggtttttgaagtagaaaatcataaaaatctagggtttttatgaattgttttgaagaaaaattgcaaaaattccacaaaatggaagaaaaataactttggaagcgcatttttcgcaaaaaaaccgaaatttttgcgtaaaatttcaaaattgcaataaaaattccacaaacatcaaatttcttaaattttttataaaaaattggatggaaacactcttgaatttagaaaaaaaatcagtttttctcatctaaaacttcaaaatttcggtgtaatcccattaaaattgccacaaaattcggaaatttcacctgaaatagagtgaataatttaaaatgttcagaaattcatattttgtcattttaaaagcattaaaacaaatcaaaaatctattttttttggttggaaaagttcaaaattctggagaaattacattaaaattttcgtaaaatatggtaaataaacgaaaatgttgagaaattaaagaaaagttacaatttttagcttaaaaattcaacattttgaggaaatgccacctaaaaaagtgactaatcgaaaatgttgagaaattaaaattgccaccatttatttatataaactactctaaaattacaattttcatgttaaaaattaataaaaaatctactttttccaaactacagtaaccttaccgtatacctacagtacctgaacattgccccccaccagctcccaacccaatacctcctcaaaaacttacacctcaatttttcataaactacagtaaccctaccaaaaaagcacaaaaaaaaatctacattcattttccaacaattttcaatattttcagGACCCAAACGAGAAGCGTGACCACATGGTCCTCCTCGAGTTCGTCACCGCCGCCGGAATCACCCACGGAATGGACGAGCTCTACAAGCCTGCAATGCAATGCGTGAGGTCATCTCTATCCACGTCGGACAAGCCGGAGTCCAAATCGGAAACGCCTGCTGGGAGCTCTACTGCCTCGAGCACGGAATCCAGCCCGATGGAACCATGCCAACTCAATCAACGAACGAGGGAGAGTCGTTCACCACTTTCTTCTCAGACACCGGATCCGGCCGTTACGTTCCAAGATCCATCTTCGTCGATCTCGAGCCAACTGTCGTTGACGAGATTCGCACTGGAACCTACAAGAAGCTCTTCCATCCAGAGCAGATGATCACCGGAAAGGAAGACGCCGCTAACAACTACGCTCGTGGACACTACACCGTCGGAAAGGAGCTCATCGACACCGTCCTCGACAGGATCCGTCGTCTCGCTGATAACTGCAGTGGACTCCAAGGATTCTTCGTCTTCCACTCCTTCGGAGGAGGTACCGGATCCGGATTCACTTCGCTTCTTATGGAACGTCTTTCCGTCGACTACGGAAAGAAGTCCAAGCTCGAGTTCTCCATCTACCCAGCTCCACAGGTCTCAACCGCCGTCGTTGAGCCATACAACTCGATCCTCACCACCCATACCACCTTGGAGCACTCCGACTGCGCCTTCATGGTCGATAACGAGGCCATCTACGACATCTGCCGCAGAAACTTGGATGTTGAGCGACCAAGCTACACCAACCTCAACAGAATCATCTCCCAGgtttgttgagctcaatttgattgttgtattctaatgttctctttttacagGTTGTCTCCTCAATCACTGCTTCCTTGAGATTCGATGGAGCCCTCAACGTTGATCTCAACGAGTTCCAGACCAACTTGGTGCCATACCCAAGAATTCACTTCCCATTGGCCGCCTACACTCCACTCATCTCTGCTGAGAAGGCCTACCACGAGGCTCTGTCCGTCAGCGACATCACCAATAGCTGCTTCGAGCCGGCTAACCAGATGGTCAAGTGTGATCCACGTCACGGAAAGTACATGGCTGTGTGCCTCTTGTACAGAGGAGACGTCGTTCCAAAGGACGTTAACACCGCCATCGCTGCAATCAAGACCAAGAGAACCATCCAATTCGTCGATTGGTGCCCA**GAG**GGATTCAAGGTCGGAATCAACTACCAGCCACCAACTGTTGTGCCAGGAGGTGATCTTGCCAAGGTGCCACGCGCCGTCTGCATGCTCTCCAACACTACCGCCATCGCTGAGGCCTGGTCTCGTCTCGACTACAAGTTCGACTTGATGTACGCCAAGCGTGCCTTCGTCCACTGgtatgttgcccgttaaactatacttttcaaatattcaatgtgtttcttttcagGTACGTCGGAGAAGGTATGGAGGAAGGAGAGTTCACCGAGGCTCGTGAGGACTTGGCTGCTCTCGAGAAGGACTACGAAGAGGTCGGAGCTGACTCCAACGAGGGAGGAGAAGAGGAGGGAGAGGAGTACTAGCGCGTCGCGTAAaTAAaTAA

GFP optimized for germline expression is highlighted in green. tba-2(T349E) is colored red. T349E mutation is emphasized in bold text.
